# Agro-waste as a substrate for the production of pullulanase by *Penicillium viridicatum* under solid-state fermentation

**DOI:** 10.1038/s41598-022-16854-4

**Published:** 2022-07-25

**Authors:** Vijay Kumar, Bindu Naik, Megha Choudhary, Akhilesh Kumar, Naresh Khanduri

**Affiliations:** 1 Himalayan School of Biosciences, Swami Rama Himalayan University, Swami Rama Nagar, Jolly Grant, Dehradun, Uttarakhand 248016 India; 2 Department of Life Sciences, Food Technology, Graphic Era Deemed to be University, Dehradun, 248002 India

**Keywords:** Biotechnology, Industrial microbiology

## Abstract

One of the key enzymes utilized in the food industry is pullulanase. But its major drawbacks are its low yield and high production costs. In this regard, the current research aims to screen agro-waste substrates for optimal pullulanase production in solid-state fermentation. Of various agro-wastes used as a substrate, the maximum enzymic activity (9.74 U/gds) was observed in a medium based on 5 g of green gram husk and incubated for 3 days at 30 °C. The effects of 16 different nutrients on the yield of pullulanase production were studied using the Plackett–Burman experimental design. The incorporation of FeSO_4_, MnSO_4_, and MgSO_4_ into the pullulanase production medium significantly increased the yield and showed a 5.7-fold increase (56.25 U/gds) in comparison with the unoptimized media. The Box-Behnken experimental design was used to study the effect of interactions between Fe^2+^, Mg^2+^, and Mn^2+^ on the production of pullulanase. Box-Behnken showed a 1.1-fold increase (62.1 U/gds) in pullulanase production. The total increase in yield after all optimization was 6.37-fold. The present study reports for the first time the applicability of green gram husk as a potent substrate for pullulanase production by *Penicillium viridicatum*.

## Introduction

Waste management is a key issue for the agro-based industry. They are accountable for polluting the air and water resources as well as having severe impacts on human health because of their disposal practices. Various chemical treatments have traditionally been employed to handle solid waste. In recent times, more emphasis has been given to the biological conversion of these agro-wastes into useful products. Reports indicate that fungi can break down these complex organic compounds into simpler ones for their energy requirements^1^. Agro-wastes are a rich source of carbon that can be used to produce both microbial biomass and metabolites. It can act as a cheaper fermentation medium for lowering the cost of enzyme production^2^.

Advances in industrial biotechnology have the potential to make agro-industrial waste more economically valuable. Rice bran and wheat bran are important byproducts of the rice and wheat processing industries, respectively. These two byproducts can be effectively used to make a variety of high-value items^3^**.** *Phaseolus vulgaris* (local red kidney beans), *Pistia stratiotes* (water cabbage), *Eichhornia crassipes* (water hyacinth), and *Ipomoea batatas* (sweet potato) were identified as novel substrates to produce pullulanase^4^. Sugarcane bagasse, banana peel, rice bran, wheat bran, mausami peel, orange peel, legume husks, and other agro-industrial wastes have all been employed as substrates to produce biocatalysts^1,5–9^. Green gram husk is rich in nutrients such as proteins (7.18%), fats (2.1%), carbohydrates (60%), fiber (18.6%), iron 23.78 mg/100 g, calcium (400 mg/100 g), phosphorous (356.55 mg/100 g), zinc (2.90 mg/100 g), and manganese (2.28 mg/100 g)^10^. Prakasham et al*.*^11^ reported maximum production of protease (9550 U/g biomass) by *Bacillus* species in green gram substrate. However, Shivasharanappa et al.^12^ reported lower production of protease in green gram husk as compared to red gram and Bengal gram husk. A similar finding was reported by Chmimata et al*.*^13^ for amylase production by *Aspergillus* species. But no data is available for pullulanase production in SSF using green gram husk as substrate by *Penicillium* species.

Among the various biocatalysts, pullulanase is one of the most important enzymes, catalyzing the α*-*1,6-glucosidic linkages to produce products like panose, maltotriose, and maltose. It also degrades starch to produce maltotriose, maltose, amylose, and amylopectin as the main products^8^. These products have been used in various food industries. In the present study, the selection of agro-waste as a substrate and nutrient optimization for the best substrate in solid-state fermentation was performed using a statistical approach. To the best of our knowledge, pullulanase production from green gram husk by *Penicillium viridicatum* under SSF has been attempted for the first time.

## Materials and methods

### The microorganism, substrate, and inoculum

The fungi used in this investigation were taken from the previous study^9^. This isolate is most closely related to *Penicillium viridicatum*^9^. The inoculum was made using the procedure reported by Francis et al*.*^14^. Different agro-wastes such as wheat bran, the husk of green gram, red gram, black gram, banana peel, and mausami peel were screened for the optimum production of pullulanase. The substrates were procured from the local market. These were washed, dried, and coarsely grounded (mesh size 2–3 mm). The inoculum of 6.42 log CFU/gds (colony-forming unit/gram dry substrate) was added to each flask with a final moisture content of 69.9%. The flasks were incubated for 72 h in a shaking flask incubator with an air blower at 28.62 ± 0.5 °C. Each substrate was tested in triplicates.

### Screening of agro-waste for optimum production of pullulanase

Salts such as 0.2% MgSO_4_, 1% KH_2_PO_4,_ 0.2% NaCl, and 1% (NH_4_)_2_SO_4_, were added to 5 g of each substrate. The inoculum of 6.42 log CFU/gds^7^ (colony-forming unit/gram dry substrate) was added to each flask with a final moisture content of 69.9%. All the flasks were then autoclaved at 121 °C for 15 min. The flasks were incubated for 72 h in a shaking flask incubator with an air blower at 28.62 ± 0.5 °C. Each substrate was tested in triplicates The substrate that produced the highest enzyme activity was chosen for further studies.

### Plackett–Burman design for selection of nutrients for optimum production of pullulanase

Sixteen independent nutrient variables such as peptone, yeast extract, urea, (NH_4_)_2_SO_4_, K_2_HPO_4_, CaCl_2_, MnCl_2_, FeSO_4_, CuSO_4_, MnSO_4_, MgSO_4_, KCl, ZnSO_4_, NaCl, KHSO_4_, NaNO_3_, and Na_2_CO_3_ were screened by Plackett–Burman to find the most effective nutrient on the yield of pullulanase. Then the most effective factors were further optimized with the Box-Behnken design by using Design expert software version 10.1. The concentration of the sixteen independent nutrient variables screened by PBD has been given in Table 1.

**Table 1:** Concentration of the sixteen independent nutrient variables screened by Plackett–Burman.

SN	Nutrients	− 1 (%) (g/g dry substrate)	+ 1 (%) (g/g dry substrate)
1	Peptone	0.1	0.2
2	Yeast extract	0.1	0.2
3	NH_4_SO_4_	0.1	0.2
4	Urea	0.1	0.2
5	KH_2_PO_4_	0.1	0.2
6	Na_2_CO_3_	0.1	0.2
7	CuSO_4_	0.05	0.1
8	FeSO_4_	0.05	0.1
9	CaCl_2_	0.05	0.1
10	MnSO_4_	0.05	0.1
11	MgSO_4_	0.05	0.1
12	KCl	0.05	0.1
13	ZnSO_4_	0.05	0.1
14	NaCl	0.05	0.1
15	KHSO_4_	0.1	0.2
16	NaNO_3_	0.1	0.2

### Box-Behnken design at three levels for selected nutrients

The concentrations of the three independent factors that exhibited a significant effect (FeSO_4_, MnSO_4_, MgSO_4_) on pullulanase production were optimized at three levels by the Box-Behnken design. The numerical optimization was carried out using the previously outlined approach^15^. The studied critical nutrients and their actual and coded values are given in Table 2.

**Table 2:** Studied critical nutrients and their levels for Box-Behnken design.

Variables	Unit	Actual values			Coded values		
FeSO_4_	% (w/w)	0.2	0.3	0.4	− 1	0	+ 1
MnSO_4_	% (w/w)	0.2	0.3	0.4	− 1	0	+ 1
MgSO_4_	% (w/w)	0.2	0.3	0.4	− 1	0	+ 1

### Crude enzyme extraction and estimation of protein

The crude enzyme was extracted by flooding flasks with 1 mM phosphate buffer (pH 6.5) at room temperature (30 °C) for 15 min. after the fungus had grown to its maximal potential. The crude enzyme was separated from the substrate and biomass mixture by using a muslin cloth. To eliminate all the cells and debris, the extract was centrifuged at 4 °C for 15 min at 10,000 rpm in a cooling centrifuge^16^. The supernatant containing the crude enzyme was decanted and separated from the pellet and was used for the estimation of pullulanase activity.

### Pullulanase activity estimation

Total pullulanase activity was measured by using the 3,5-dinitrosalicylic acid (DNS) method^17^. 0.5 mL of 1% (w/v) pullulan solution was mixed with 0.1 mL of enzyme sample and 0.4 mL of phosphate buffer (pH 6.5). The reaction mixture was kept at 40 °C for 30 min. Test tubes were incubated in a boiling water bath for 5 min. after adding 1 mL of DNS reagent. The liquid was then cooled to room temperature before adding 0.5 mL of a 1% (w/v) sodium potassium tartrate solution. The final volume was increased to 5 mL by adding 2.5 mL of double-distilled water. Using a UV–Vis Spectrophotometer, absorbance was measured at 570 nm (Shimazu-UV 1800, Japan). One unit of pullulanase was defined as the quantity of enzyme that released one micromole of glucose (reducing sugar equivalent) per minute at 40 °C and pH 6.5.

## Results and discussion

### The microorganism

The fungi used in the present study were isolated, screened, and identified in the previous study^9^. The sequence was submitted to GenBank under accession number MG672442. This isolate is most closely related to *Penicillium viridicatum*^9^*.*

### Selection of agro-wastes for production of pullulanase in solid-state fermentation (SSF)

**Figure 1:**
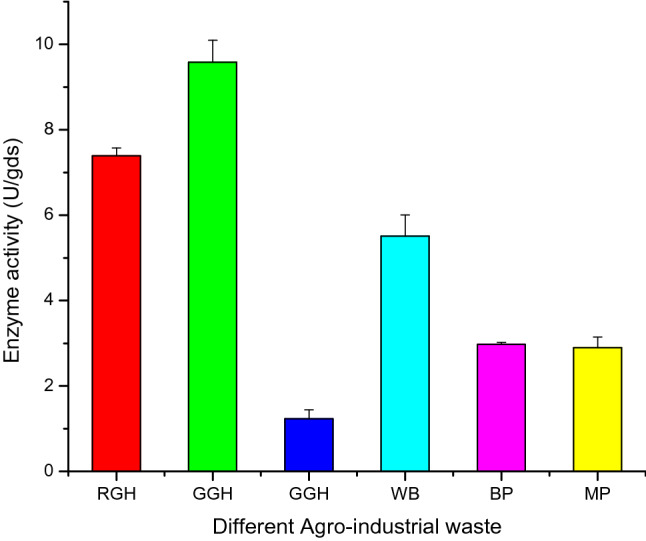
Selection of agro-based substrates for pullulanase production. RGH, red gram husk, GGH, green gram husk, BGH, black gram husk, WB, wheat bran, BP, banana peel, MP, mausambi peel; error bar indicates the average of triplicates ± standard deviation.

One of the most important variables to consider is the selection of an appropriate agricultural residue as an SSF substrate. In SSF, different substrates have been screened for high yields of enzyme production^18^. When selecting a raw material in SSF, the availability and cost of the raw material are the two most important factors that need to be considered^19^. The chosen substrate facilitates the growth and development of microorganisms along with the synthesis of metabolites. In the present study, six major agricultural waste-based substrates such as wheat bran, green gram husk, red gram husk, black gram husk, banana peel, and mausambi peel were assessed for pullulanase production. Green gram husk had the highest enzyme activity (9.7 U/gds) of all the substrates studied (Fig. 1), followed by red gram husk (7.29 U/gds) and wheat bran (5.52 U/gds). According to the findings of this study, pullulanase synthesis differed with a different kind of substrate due to differences in food supply and anchorage for growing cells. Proteins, lipids, carbohydrates, and minerals including iron, calcium, phosphorus, manganese, zinc, and copper are claimed to be abundant in the green gram husk^10^. There is no report on pullulanase production by *Penicillium* species in solid-state fermentation (SSF) using green gram husk as a substrate. Wheat bran was reported as a substrate for pullulanase production by *Aspergillus flavus* in SSF by Naik et al.^11^. The green gram husk was successfully used by Prakasham et al*.*^18^ to produce the protease (9550 U/g biomass) by *Bacillus* species, but there are no reports on pullulanase production. This is the first time that green gram husk has been used as a substrate in SSF to produce pullulanase by *Penicillium viridicatum***.**

### Screening of important nutrients for green gram husk substrate using Plackett–Burman design

**Figure 2:**
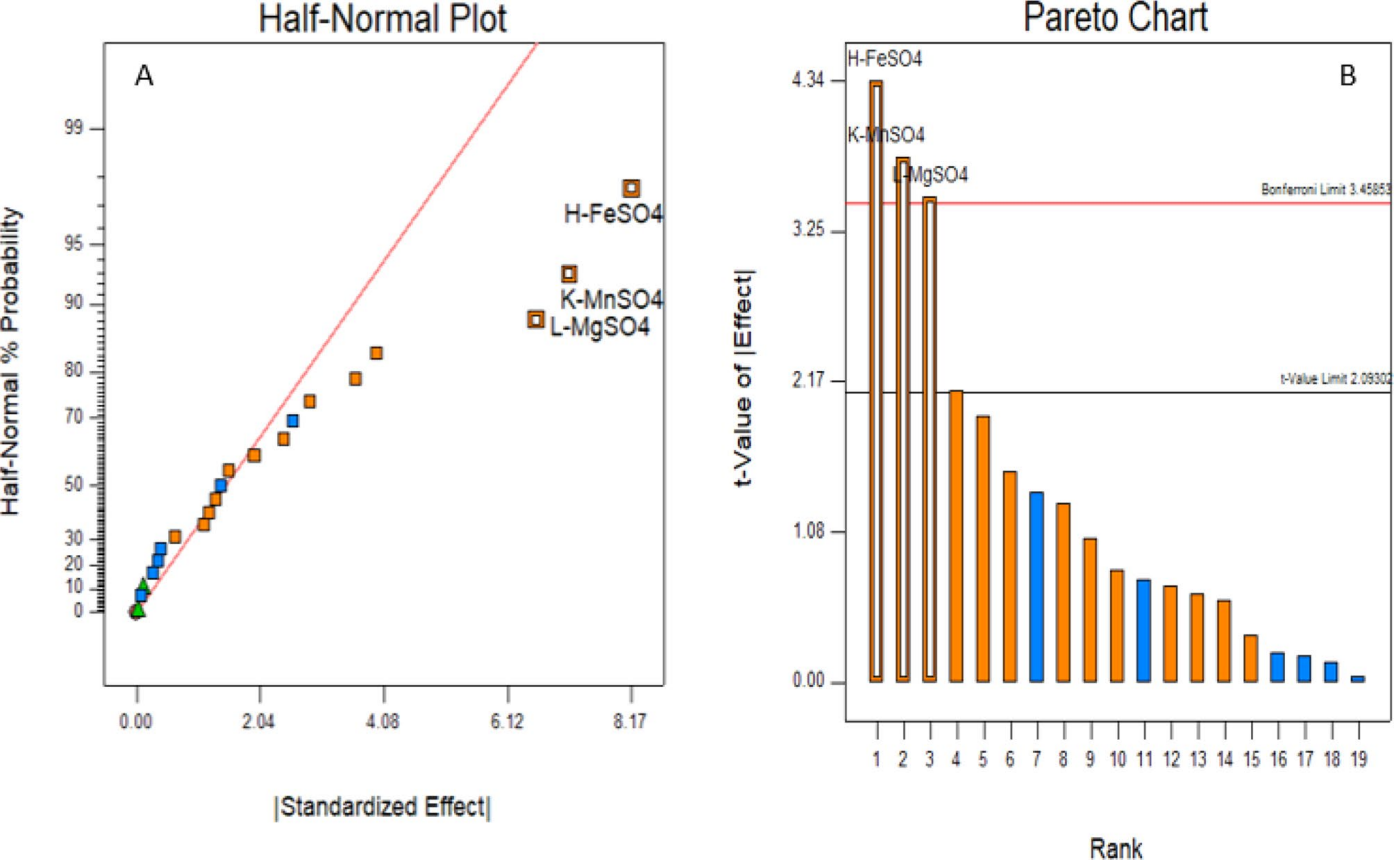
(**A**) Half-normal plot; (**B**) Pareto chart showing the effects of different variables.

Plackett–Burman design (PBD) was previously used for quick screening of different nitrogen sources, growth/product promoters, minerals, and enzyme inducers for the synthesis of alpha-galactosidase by *Aspergillus niger* in a solid-state fermentation system^20^. Using shea butter cake as the major substrate, it was employed to efficiently identify essential medium components affecting *Aspergillus niger* lipase production^21^. Similarly, PBD was used for screening nutrients for laccase production for *Bacillus* species^22^. Based on the above studies a total of 16 different nutrients and three dummy factors were used to screen the most effective nutrient to produce pullulanase by using the Plackett–Burman design. The number of experiments to be carried out based on the PBD design is n + 1, where n is the number of factors (variables). The high variables were designated as + 1 and the low variables as − 1^23,24^. The response of the 16 nutrients plus three dummy variables to the pullulanase production is given in Table 3. The results of the PBD showed that run 12 (56.25 U/gds; Units/grams of the dry substrate) had the maximum yield, followed by runs11 (51.67 U/gds), 17 (49.46 U/gds), and 18 (48.79 U/gds). The contrast coefficient (*b*) study revealed that out of 19 variables, only three variables (FeSO_4:_ b = 4.08), MnSO_4_: b = 3.57), and MgSO_4_: b = 3.30) had a significant effect on the synthesis of pullulanase. This value was low in the case of peptone, yeast extract, and urea hence not selected for further studies^14^. Moreover, the green husk is rich in proteins^10^. The rest of the sixteen variables were not selected because they did not contribute significantly to pullulanase production at the selected level of confidence. A half-normal plot demonstrating the amplitude and orientations of standardized effects of major nutrients is shown in Fig. 2A. A half-normal probability plot is a graphical tool for determining which elements are important and which are not using these ordered estimated effects. FeSO_4_ is the furthest to the right of the response line, suggesting that it has the most positive impact on *Penicillium viridicatum* pullulanase production. Similarly, the order of relevance (MnSO_4_, FeSO_4_, and MgSO_4_) of the variables influencing pullulanase production is depicted in the Pareto chart (Fig. 2B). Metal ions are essential in the production of enzymes^25^. Previously Reddy et al.^26^ reported that FeSO_4_ has a significant effect on pullulanase production by *Clostridium thermosulfurogenes* in SSF. Zhang et al.^27^ showed that ferrous ions were required for enzyme synthesis, although their absence did not affect the growth of the culture. Similarly, MnSO_4_ and MgSO_4_ have also shown a positive effect on pullulanase yield. Manganese has been shown to significantly increase the synthesis of pullulanase and there was a 1.8-fold increase in pullulanase production by using MnSO_4_ in production media^28,29^. Mn^2+^ was found to increase enzyme production in earlier studies by other researchers^30,31^. *A. flavus* produced more enzymes when MgSO_4_.7H_2_O was added to the wheat bran medium^25^. Similarly, Kokab et al*.*^32^ reported a higher yield of enzyme in SSF when the solid substrate was supplemented with MgSO_4_.

**Table 3:** Nutrient contributions to pullulanase production by Placket-Burman design matrix (randomized).

Std	Run	A	B	C	D	E	F	G	H	J	K	L	M	N	O	P	Q	R	S	T	Response: Enzyme activity (U/gds)
**Factors (coded values)**																					
13	1	1	− 1	1	− 1	1	− 1	− 1	− 1	− 1	1	1	− 1	1	1	− 1	− 1	1	1	1	40.00
7	2	− 1	− 1	− 1	1	1	− 1	1	1	− 1	− 1	1	1	1	1	− 1	1	− 1	1	− 1	41.00
2	3	− 1	1	1	− 1	− 1	1	1	1	1	− 1	1	− 1	1	− 1	− 1	− 1	− 1	1	1	43.00
11	4	1	− 1	1	− 1	− 1	− 1	− 1	1	1	− 1	1	1	− 1	− 1	1	1	1	1	− 1	41.18
6	5	− 1	− 1	1	1	− 1	1	1	− 1	− 1	1	1	1	1	− 1	1	− 1	1	− 1	− 1	43.73
19	6	1	− 1	− 1	1	1	1	1	− 1	1	− 1	1	− 1	− 1	− 1	− 1	1	1	− 1	1	38.00
15	7	1	1	1	− 1	1	− 1	1	− 1	− 1	− 1	− 1	1	1	− 1	1	1	− 1	− 1	1	29.57
18	8	− 1	− 1	1	1	1	1	− 1	1	− 1	1	− 1	− 1	− 1	− 1	1	1	− 1	1	1	39.45
9	9	1	− 1	− 1	− 1	− 1	1	1	− 1	1	1	− 1	− 1	1	1	1	1	− 1	1	− 1	30.00
17	10	− 1	1	1	1	1	− 1	1	− 1	1	− 1	− 1	− 1	− 1	1	1	− 1	1	1	− 1	38.15
10	11	− 1	1	− 1	− 1	− 1	− 1	1	1	− 1	1	1	− 1	− 1	1	1	1	1	− 1	1	51.67
4	12	1	1	− 1	1	1	− 1	− 1	1	1	1	1	− 1	1	− 1	1	− 1	− 1	− 1	− 1	56.25
20	13	− 1	− 1	− 1	− 1	− 1	− 1	− 1	− 1	− 1	− 1	− 1	− 1	− 1	− 1	− 1	− 1	− 1	− 1	− 1	21.23
8	14	− 1	− 1	− 1	− 1	1	1	− 1	1	1	− 1	− 1	1	1	1	1	− 1	1	− 1	1	38.00
16	15	1	1	1	1	− 1	1	− 1	1	− 1	− 1	− 1	− 1	1	1	− 1	1	1	− 1	− 1	35.67
12	16	− 1	1	− 1	1	− 1	− 1	− 1	− 1	1	1	− 1	1	1	− 1	− 1	1	1	1	1	40.35
3	17	1	− 1	1	1	− 1	− 1	1	1	1	1	− 1	1	− 1	1	− 1	− 1	− 1	− 1	1	47.00
1	18	1	1	− 1	− 1	1	1	1	1	− 1	1	− 1	1	− 1	− 1	− 1	− 1	1	1	− 1	47.00
14	19	1	1	− 1	1	− 1	1	− 1	− 1	− 1	− 1	1	1	− 1	1	1	− 1	− 1	1	1	37.91
5	20	− 1	1	1	− 1	1	1	− 1	− 1	1	1	1	1	− 1	1	− 1	1	− 1	− 1	− 1	39.62
22	21	0	0	0	0	0	0	0	0	0	0	0	0	0	0	0	0	0	0	0	28.54
21	22	0	0	0	0	0	0	0	0	0	0	0	0	0	0	0	0	0	0	0	25.44
23	23	0	0	0	0	0	0	0	0	0	0	0	0	0	0	0	0	0	0	0	27.33

Symbols used: A-peptone; B-Yeast extract; C-NH_4_SO_4_,; D-urea; E-KH_2_PO_4_; F-Na_2_CO_3_; G-CuSO_4_; H-FeSO_4_; J-CaCl_2_; K-MnSO_4_; L-MgSO_4_; M-KCl; N-ZnSO_4_; O-NaCl; P-KHSO_4_; Q-NaNO_3_; R- Dummy 1; S-Dummy 2; T-Dummy.

### Box-Behnken design

**Figure 3:**
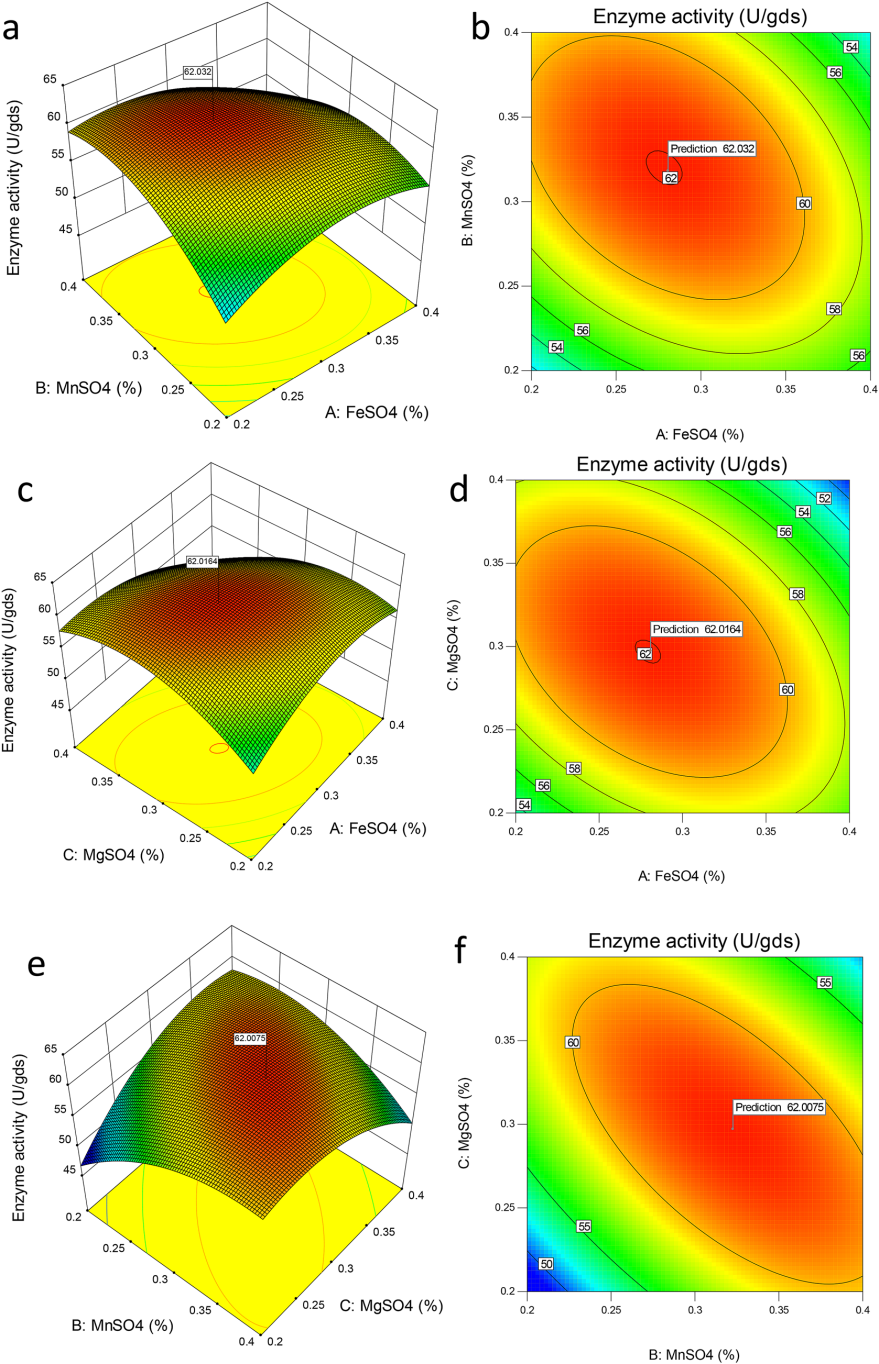
(**a**–**f**) Showing the effect of nutrients supplemented in green gram husk for pullulanase production.

Based on the findings of the PBD, the Box-Behnken design was used to get the optimal concentration of chosen nutrients. Table 4 shows the nutrient levels as well as the outcomes. The results were entered into the program, and an ANOVA was run. Equation (1) depicts the regression model that resulted from the data analysis.

1 $$ Y = + 61.84 - 1.25x_{1} + 0.45x_{2} - 0.38x_{3} - 2.76x_{1} x_{2} - 3.10x_{1} x_{3} - 5.17x_{2} x_{3} - 3.45x_{1}^{2} - 3.89x_{2}^{2} - 4.23x_{3}^{2} $$

where *Y* is the yield*,* $x_{1}$*,* $x_{2}$, $x_{3}$ and are the concentrations of FeSO_4_, MnSO_4_, and MgSO_4_ respectively. The R^2^ value of 0.9922 demonstrated that the independent variables were responsible for 99.22% of the sample variations in pullulanase output, whereas just 0.78% of the total changes were not explained by the model. The Adjusted R^2^ value of 0.9821 was likewise quite high, indicating that the model is very important. The CV value is 1.19% (a relatively low number), indicating that the studies were more accurate and efficient. The significant model terms are $x_{1}$, $x_{1} x_{2}$, $x_{1} x_{3}$, $x_{2} x_{3}$*,* $x_{1}^{2}$*,* $x_{2}^{2}$*,* and $x_{3}^{2}$ (Table 5). The 3D response surface graphs and contour plots show how critical parameters interact and provide a visual representation of where the optimum conditions are located. Figure 3a–f was created for two parameters at a time, with the other variables maintained at their maximum value. The software's numerical optimization function was used to find the optimal levels (Design-Expert). During the optimization for the response, the variables FeSO4, MnSO4, and MgSO4 were placed in their ranges while the response (enzyme activity) was set to maximum level. The best option that met all of the aforementioned criteria and had overall desirability of 0.974 was found (FeSO_4_, 0.27%; MgSO_4_, 0.29%; MnSO_4_, 0.31%). Figure 3a,b depicts the interaction between MnSO_4_ and FeSO_4_ on the production of pullulanase. When the concentrations of MnSO_4_ and FeSO_4_ were increased, the curve of the graph showed a strong positive interaction with pullulanase production. The effect of MgSO_4_ and FeSO_4_ on pullulanase production was seen in Fig. 3c,d. As was seen in the graph, increasing the MgSO_4_ concentration enhanced pullulanase production. In the case of FeSO_4_, a similar trend has been found. The interaction of MgSO_4_ and MnSO_4_ is shown in Fig. 3e,f, and optimal production may be attained at lower concentrations. This finding is in accordance with the cited literature^33,34^. In these publications, MgSO_4_ at higher levels has been reported to reduce the production of enzymes. It has been reported previously that FeSO_4_, MgSO_4_, and MnSO_4_ have a positive impact on enzyme production^35–38^. It may be due to activation, stability, simulation by these salts, and possible utilization of sulphate in protein synthesis^39,40^. In a study done by Alariya et al*.*^41^ it was reported that manganese sulfate was the most suitable sulfate source**.** According to a study by Zhang et al.^42^ Fe^2+^ was found to be essential for enzyme production, although there was hardly any effect seen on cell growth in the absence of this ion.

**Table 4:** Box Behnken design showing effect of various factors on pullulanase production.

Run	FeSO_4_	MnSO_4_	MgSO_4_	Actual enzyme activity (U/gds)	Predicted enzyme activity (U/gds)
1	0.4	0.4	0.3	50.83	50.93
2	0.3	0.3	0.3	62.3	61.84
3	0.3	0.3	0.3	62.4	61.84
4	0.3	0.2	0.4	57.46	58.06
5	0.3	0.3	0.3	61.00	61.84
6	0.4	0.3	0.4	49.70	49.42
7	0.2	0.3	0.4	58.63	58.12
8	0.3	0.4	0.2	60.32	59.71
9	0.4	0.3	0.2	55.89	56.39
10	0.2	0.4	0.3	58.63	58.95
11	0.2	0.2	0.3	52.64	52.54
12	0.3	0.4	0.4	48.44	48.61
13	0.3	0.3	0.3	61.4	61.84
14	0.3	0.3	0.3	62.1	61.84
15	0.4	0.2	0.3	55.89	55.56
16	0.2	0.3	0.2	52.41	52.68
17	0.3	0.2	0.2	48.67	48.49

**Table 5:** Regression coefficients and statistical significance for the quadratic model.

Source	Sum of squares	Df	Mean square	F value	*p*-value prob > F
Model	402.33	9	44.70	98.67	< 0.0001*
A-FeSO_4_	12.50	1	12.50	27.59	0.0012*
B-MnSO_4_	1.58	1	1.58	3.50	0.1037**
C-MgSO_4_	1.17	1	1.17	2.58	0.1520**
AB	30.53	1	30.53	67.38	< 0.0001*
AC	38.50	1	38.50	84.99	< 0.0001*
BC	106.81	1	106.81	235.77	< 0.0001*
A^2^	50.22	1	50.22	110.86	< 0.0001*
B^2^	63.67	1	63.67	140.55	< 0.0001*
C^2^	75.29	1	75.29	166.20	< 0.0001*
Residual	3.17	7	0.45		
Lack of Fit	1.68	3	0.56	1.50	0.3427
CV:1.19	R^2^: 99.2	Adjusted R^2^: 98.2%		Predicted R^2^:92.8%	

*Symbols used*: *, Significant terms; **, non-significant terms; AB-interactive term for FeSO_4_ and MnSO_4_; AC-interactive term for FeSO_4_ and MgSO_4_; BC-interactive term for MSO_4_ and MgSO_4_.

### Model validation

Validation of the model was carried out in the model's predicted conditions. The best-applied levels for each variable in the substrate green gram were 0.30% for FeSO_4_, MnSO_4_, and MgSO_4_.The yield was 9.7 U/gds before nutrient optimization. Using the optimized medium ingredient concentration (0.27% FeSO4; 0.29% MgSO4; 0.31% MnSO4), the estimated pullulanase yield was 62.4 U/gds. Additional triplicate tests with the improved media were performed to confirm the model's prediction. The current study produced a maximum pullulanase activity of 62.4 U/gds. Through predicted and tested values, the validity and feasibility of optimum points were confirmed. There was a 6.4-fold increase in total yield.

## Conclusion

It can be concluded that green gram husk is suitable as a substrate to produce pullulanase in SSF by *Penicillium* sp. When this substrate was further supplemented with FeSO_4_, MnSO_4_, and MgSO_4_, the total yield increased by 6.4 times. This is the first report on pullulanase production by *Penicillium* species using green husk as in substrate in SSF and its nutrient optimization.

## Data Availability

The datasets used and analyzed during the present study are available from the corresponding author on reasonable request.

## Abbreviations

U/gds: Units per gram dry substrate
PBD: Plackett–Burman design
rpm: Revolution per minute
VC: Coefficient of variation
w/w: Weight by weight
Df: Degree of freedom
Prob: Probability
SD: Standard deviation
